# β-Actin as an Endogenous Control Gene in Real-Time PCR for Detection of West Nile and Usutu Virus in Mosquitoes

**DOI:** 10.3390/microorganisms13112518

**Published:** 2025-10-31

**Authors:** Jeanne Lai, Carlotta Tessarolo, Elisabetta Ercole, Marina Gallo, Monica Lo Faro, Claudia Palmitessa, Valerio Carta, Alessio Ferrari, Alessandra Favole, Mattia Begovoeva, Francesco Ingravalle, Simone Peletto, Nicolò Francesco Fiscella, Roberta Irelli, Eugenia Ciarrocchi, Walter Martelli, Andrea Mosca, Giulia Cagnotti, Cristina Casalone, Cristiano Corona

**Affiliations:** 1 Istituto Zooprofilattico Sperimentale del Piemonte, Liguria e Valle d’Aosta (IZSPLV), 10154 Turin, Italy; jeannelai3@gmail.com (J.L.); simone.peletto@izsplv.it (S.P.); walter.martelli@izsplv.it (W.M.);; 2 Istituto Zooprofilattico Sperimentale dell’Abruzzo e del Molise, 64100 Teramo, Italy; 3 Istituto per le Piante da Legno e l’Ambiente (IPLA SpA), 10132 Turin, Italy; 4 Department of Veterinary Science, University of Turin, 10095 Grugliasco, Italy

**Keywords:** West Nile virus, Usutu virus, β-Actin (ACTB), mosquitoes borne virus surveillance, internal control, housekeeping gene

## Abstract

Mosquito-borne viruses like West Nile virus (WNV) and Usutu virus (USUV) present growing public health concerns, especially with climate change and expanding vector ranges. This study describes the development and validation of a duplex Real-Time RT-PCR assay targeting β-actin (ACTB) mRNA as an endogenous control and a conserved 92 bp region shared by WNV and USUV genomes. Degenerate primers for ACTB ensure RNA extraction quality and PCR performance while enabling simultaneous detection of both viruses. A total of 1002 mosquito pools collected in Piedmont, Italy, during the 2024 vector season under the National Surveillance Plan for Arboviruses (PNA), were tested. The assay showed 100% accuracy—ACTB mRNA was detected in all pools, and six pools tested positive for WNV or USUV (three each). Diagnostic specificity was confirmed on 40 horse and bovine serum samples. Sanger sequencing confirmed ACTB identity across multiple mosquito species. The assay also demonstrated reproducibility across different operators and thermocyclers. The limit of detection (LOD) evaluation showed that the assay is capable of detecting viral RNA at very low concentrations, confirming its high analytical sensitivity. The duplex RT-PCR here developed is a reliable, sensitive, and specific tool for arbovirus surveillance, combining pathogen detection with internal quality control of RNA extraction and amplification, thus improving early warning and rapid response to mosquito-borne disease threats.

## 1. Introduction

Climate change in recent years has impacted ecosystems worldwide. As temperatures rise, mosquito activity intensifies, thus favouring the transmission of several pathogens, including those belonging to the Orthoflavivirus genus, which cause diseases in humans and other animals, both wild or domestic [1]. As a result, considering Orthoflavivirus, the number of human cases of West Nile virus (WNV) infections has increased not only in the United States of America (USA) but also in Europe, particularly in Central and Mediterranean Europe [2,3,4]. Orthoflaviviruses transmitted by mosquitoes have been circulating in Europe for more than 20 years. Usutu virus (USUV) was first identified in Austria in 2001 and later retrospectively detected in samples from Italy dating back to 1996 [5]. West Nile virus (WNV) is one of the most widespread arboviruses worldwide and has been present in Europe since 1996. Both USUV and WNV have now been detected in many parts of Europe [6]. The European Centre for Disease Prevention and Control (ECDC) has reported nearly 700 human cases, with the highest frequency of WNV infections in Italy (i.e., 336 human cases of WNV infections) [7]. In this context, the Italian National Surveillance Plan (Italian National Institute of Health, PNA 2020–2025, [8]) is a nationwide collaboration that enables all levels of public health (local and national) to share health information to monitor, control, and prevent the occurrence and spread of state-reportable and nationally notifiable infections [9]. Surveillance programmes are specifically designed to monitor West Nile virus, Usutu virus, Chikungunya, Dengue, Tick-borne encephalitis (TBE), and neuroinvasive infections caused by Toscana virus (TOSV). PNA prescribes the systematic placement of specialized traps at strategic sites across the territory to enable comprehensive mosquito surveillance. Once captured, female mosquitoes are grouped into pools based on species and trapping site. The presence of WNV and USUV pathogens is evaluated by extracting RNA from mosquito pools, and samples are subjected to biomolecular analyses, such as Real-Time PCR, to detect the presence of viruses. To ensure the reliability and accuracy of the results, biomolecular methods typically involve the simultaneous amplification of the target sequence and a housekeeping gene whose function is to verify the quality of RNA extraction and confirm the proper functionality of the PCR. Compared to commercial exogenous controls that have shown disappointing performance in laboratory settings when applied to mosquito pools [10], housekeeping genes are constitutive genes whose products are involved in basic cellular functions. These genes are ubiquitously expressed in all cells of an organism, regardless of environmental conditions [11]. Dozens of housekeeping genes have been described [12], including those coding for ribosomal RNA, enzymes (cyclic c-oxidase, glyceraldehyde-3-phosphate dehydrogenase), structural proteins (actin, dynein), transcription factors, and other types of proteins (ubiquitin, receptors). β-actin (ACTB), a member of the actin gene family, is one of the most used housekeeping genes. ACTB is a ubiquitously expressed cytoplasmic protein involved in cytoskeleton maintenance and cellular motility [13]. This gene is highly conserved among eukaryotic species [14] and therefore represents a promising target for designing broad-spectrum molecular assays. Various molecular assays have been developed to detect and amplify ACTB mRNA in a species-specific manner [15,16,17]. Moreover, Piorkowski et al. [18] described a novel PCR protocol targeting the ACTB gene from various animal species through the use of degenerated primers able to amplify the ACTB sequence in a panel of vertebrate and invertebrate cell lines: two primers (forward and reverse) targeting ACTB and a probe were designed to match conserved nucleotide sequences within exon V of the ACTB gene. However, studies conducted so far were performed on in vitro cells, but there are a few comparisons with field samples. The purpose of the present project was to evaluate the applicability of these degenerate primers in vivo in mosquito samples captured during surveillance plans in order to improve the detection of mosquito-borne pathogens, avoiding false negative results. Therefore, a Real-Time Multiplex RT-PCR assay was developed by combining the use of degenerate primers (and probe) for ACTB mRNA with specific primers able to detect a common mRNA region of WNV and USUV spanning nucleotides 10,533–10,625 of the WNV 3—noncoding region [19]. Furthermore, the overall quality of the reaction process using mRNA of the ACTB gene as a housekeeping gene has been evaluated.

## 2. Materials and Methods

### 2.1. Samples

All the female mosquitoes collected during the PNA’s capture programme in 2024 (*n* = 1002 mosquito pools) were included in this study. Analyzed samples were representative of all mosquito species present in Piedmont region and belonging to the genera *Aedes*, *Anopheles*, *Culex*, *Ochlerotatus*, and *Culiseta*. Samples from all Piedmont provinces were analyzed to fully cover their varying distribution across the territory and were also categorized by trap localization and date of capture. Table 1 shows data on the number of pools and individual mosquitoes analyzed for each species divided by province of capture. Additionally, 40 serum samples (30 from horses and 10 from bovines) were used as negative controls for the presence of ACTB mRNA. Among the 30 horses’ serum, obtained from an interlaboratory proficiency test made every year to evaluate the performance of the laboratory in detecting WNV and USUV, 6 were positive for WNV Lineage 1, 4 were positive for WNV Lineage 2 and 4 for USUV (all the results were officially confirmed by the National Reference Centre for Arboviroses, CESME, Teramo, Italy). All samples were stored at −80 °C awaiting further biomolecular analysis.

### 2.2. RNA Extraction and Thermocycling Conditions

RNA extraction from mosquito pools was performed using Qiagen RNeasy Minikit (Qiagen, Hilden, Germany) with Qiacube automatic extractor (Qiagen). Briefly, mosquito pools were homogenized in 1X Phosphate-Buffer Saline (PBS) (600 µL for pools of up to 29 mosquitoes, 1.200 µL for pools of 30–100 mosquitoes) and centrifuged at 14.000× *g* for 10 min at 4 °C. 150 µL of supernatant were then transferred into 2 mL Eppendorf tubes previously filled with 600 µL of RNA Lysis Buffer (RLT) supplied by the kit and put into Qiacube automatic extractor with all the reagents, columns and plastic supports required. The extracted RNA was then stored at −80 °C in order to preserve its integrity.

Likewise, RNA extraction from serum samples was performed manually using QIAamp Viral RNA Mini Kit (Qiagen) following the manufacturer’s instructions. Extracted samples were stored at −80 °C for subsequent analysis.

All samples were amplified with a duplex Real-Time RT-PCR, combining the use of degenerate primers and a Taqman probe targeting ACTB mRNA sequences (as reported by Piorkowski et al. (2014) [18] with a couple of primers (hereafter “WNTang”) and the respective Taqman probe for the detection of a conserved 92 bp noncoding region. This region is common for both WNV Lineage 1 and 2 and USUV [19]. Primer and probe sequences (provided by Metabion International AG, Germany) are listed in Table 2. For each sample, the duplex Real-Time RT-PCR reaction consisted of 0.25 µL QuantiTect Probe RT (Qiagen), 12.5 µL 2x QuantiTect Probe RT-Mix buffer (Qiagen), 0.5 µL ACTB primers F/R 50 µM, 0.5 µL ACTB probe (10 µM), 1.25 µL WNTang primers F/R 20 µM, 0.25 µL WNTang probe (20 µM) and 3 µL of RNase-free water. The thermocycling conditions used were optimized on a CFX 96 C1000 Touch Thermal Cycler (Bio-Rad, Hercules, CA, USA) and on a CFX Opus 96 (Bio-Rad) and are reported in Table 3.

WNV and/or USUV positive samples were subjected to two confirmatory Real-Time RT-PCR: one specific for WNV Lineage 1 and 2 and one specific for USUV. Briefly, for WNV confirmation the duplex Real-Time RT-PCR reaction consisted of 0.25 µL QuantiTect Probe RT (Qiagen), 12.5 µL 2x QuantiTect Probe RT-Mix buffer (Qiagen), 0.5 µL WN primers F/R (20 µM), 0.5 µL WNV Lineage 1 and Lineage 2 probe (10 µM) and 5.25 µL of RNase-free water. For USUV the reaction was made of 0.25 µL QuantiTect Probe RT (Qiagen), 12.5 µL 2x QuantiTect Probe RT-Mix buffer (Qiagen), 2.25 µL USUTU primers F/R (10 µM), 0.25 µL USUTU probe (20 µM) and 2.5 µL of RNase-free water. Probe and Primer sequences are reported in Table 4, while the thermocycling profile is the same as in Table 3 [20,21].

### 2.3. Sequencing

To confirm the specificity of the primers for the actin gene in mosquitoes, 10 samples from various species were analyzed by Sanger sequencing. Amplicons were purified with the High Pure PCR Product Purification Kit (Roche, Basel, Switzerland). The cycle sequencing reaction for dye terminator insertion for Sanger sequencing was carried out according to the protocol of the BigDye^®^ Terminator v1.1 Cycle Sequencing Kit (Applied Biosystems, Waltham, MA, USA): 2 µL BigDye^®^ Terminator v1.1 Ready Reaction Mix, 1 µL 5X Sequencing Buffer, 0.32 µL 100 µM primer, 4.68 µL ultrapure H_2_O, and 2 µL amplicon. PCR primers were used for sequencing, and the temperature profile used for the reaction was as follows: 96 °C for 1 min; 25 cycles of 96 °C for 1 min, 50 °C for 5 s, 60 °C for 4 min. The reaction was purified using the GE Healthcare illustrates™ AutoSeq G-50 Columns kit (GE Healthcare, Chicago, IL, USA) to remove dye terminators and then analyzed on the SeqStudio sequencer (Applied Biosystems). Chromatograms were analyzed with Sequencing Analysis v. 5.2 software (Applied Biosystems) for base calling and with SeqMan software v18.1 (Lasergene, DNA STAR, Madison, WI, USA) for forward and reverse sequence alignment, primer deletion and manual correction if required. The nucleotide sequences thus obtained were used as queries in the nt database search using Blastn tool (NCBI, National Center for Biotechnology Information, Bethesda, ML, USA).

### 2.4. Statistical Analysis

#### 2.4.1. Accuracy

The accuracy of the developed method was calculated through the evaluation of diagnostic sensitivity (se) and specificity (sp) with the 95% confidence intervals (Clopper-Pearson exact method [22]). Diagnostic sensitivity is the ability of the assay to correctly identify positive samples among all tested samples. Diagnostic specificity is the assay’s ability to correctly identify negative samples.

As reported in the previous section, 1002 mosquito pools served as positive samples, and 40 serum samples (30 from horses and 10 from bovines) served as negative samples.

The sample size was defined based on both the expected sensitivity (100% with an error less than 0.5%) and specificity (100% with an error less than 10%), as well as the quantity of samples and Real-Time PCRs available.

#### 2.4.2. Reproducibility

The reproducibility of the biomolecular method was assessed by evaluating the effect of two factors: the operator who performed the analysis and the thermocycler applied in the analysis.

Two different operators analyzed 48 samples: 45 WNS/USUV negative and ACTB positive samples; 2 WNS/USUV positive and ACTB positive samples; 1 WNS/USUV negative and ACTB negative sample) with two distinct thermocyclers.

In order to evaluate the reproducibility of the biomolecular method, we estimated the Cohen’s kappa [23] between the results obtained in the two runs.

#### 2.4.3. Limit of Detection (LOD)

To evaluate the LOD of the method, we used heat-inactivated (2 h at 56 °C) viral lysates obtained from viral cultures and kindly provided by Dr Morelli from the National Reference Centre for Arboviroses (CESME, Teramo, Italy). Specifically, to determine the LOD of West Nile virus Lineage 1, a viral lysate from strain E101 (10^4.8^ TCID_50_/mL, batch 1/23) was used. For West Nile virus Lineage 2, the lysate from strain B956 (10^4.5^ TCID_50_/mL, batch 3/24) was used, and for Usutu virus the lysate from strain 939 (10^5.72^ TCID_50_/mL, batch 1/25) was used. Tenfold serial dilutions were prepared in two different matrices: (I) mosquito homogenate in 1X PBS obtained from pools composed of 1–29 mosquitoes and (II) mosquito homogenate in 1X PBS obtained from pools composed of 30–100 individuals. Subsequently, each sample was extracted and subjected to biomolecular analysis as described above.

## 3. Results

**Figure 1:**
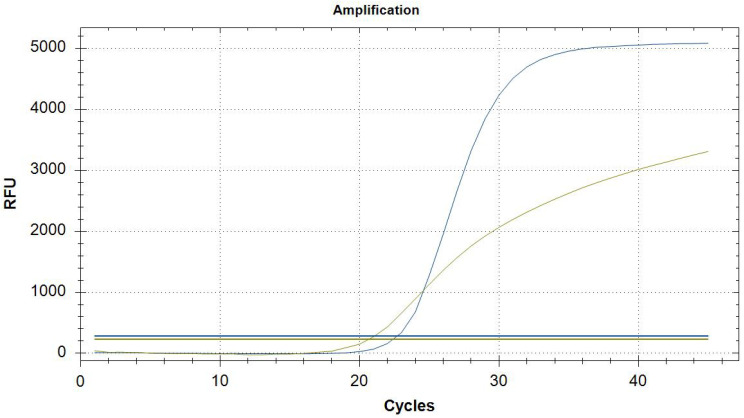
Positive sample for ACTB (in green) and the common region of 92 bp between WNV and USUTU (in blue).

All 1002 mosquito pools (summarized in Table 1) collected throughout the vector season were analyzed using our in-house duplex Real-Time RT-PCR assay. This assay was specifically designed to simultaneously detect both West Nile virus (Lineage 1 and Lineage 2) and Usutu virus, as well as ACTB (β-actin) as an endogenous control gene. The amplification of ACTB served as an internal control to confirm the quality of RNA extraction and the efficiency of the amplification process. Furthermore, by testing pools of different mosquito counts, we aimed to assess both the potential impact of reaction inhibitors (especially in pools with a high number of mosquitoes) and the sensitivity of our assay in detecting ACTB, even in pools containing only a single mosquito. Specifically, 342 pools contained fewer than 5 mosquitoes each, 311 pools included between 6 and 25 mosquitoes, 103 pools consisted of 26 to 50 mosquitoes, 61 pools contained between 51 and 75 mosquitoes, and 184 pools comprised 76 to 100 mosquitoes. In all tested samples, the ACTB segment was successfully amplified, while in six samples, the 92 bp common region shared by WNV and USUV was detected simultaneously with the ACTB amplicon (Figure 1).

Two different Real-Time PCRs, following a protocol in use in the laboratory, were performed on the six positive samples for both ACTB and the 92 bp common region shared by WNV and USUV, to discriminate the presence of WNV Lineage 1 and/or Lineage 2 and/or USUV: three pools resulted positive for the presence of WNV Lineage 2 and three pools for the presence of USUV. Positive samples, in accordance with the guidelines of PNA, were sent to the National Reference Center for Exotic Animal Diseases (CESME, Teramo, Italy) for confirmatory tests. All positive pools consisted of mosquitoes belonging to the Culex pipiens species. Furthermore, the presence of the ACTB gene was confirmed in all the analyzed mosquito genera and species, with no differences among genera or species within the same genus. Notably, the amplification of ACTB mRNA was also observed in pools consisting of individual mosquito specimens.

Among the 30 horse sera, obtained from an interlaboratory proficiency test, using the duplex Real-Time PCR developed here, 6 were confirmed positive for WNV Lineage 1, 4 for WNV Lineage 2 and 4 for USUV, as expected. No amplification of ACTB was observed either in these samples or in the 10 bovine serum samples tested.

The threshold cycle (Ct) values for the duplex RT-PCR ranged between 20 and 23 for ACTB and between 21 and 32 for WNV/USUV common sequence. Values of Ct > 40 cycles were considered not reliable.

Ten samples, randomly selected from various species, underwent Sanger sequencing to verify the specificity of the primers for the ACTB gene in mosquitoes, which confirmed 100% similarity of the obtained sequences with mosquito ACTB gene sequence records present in the NCBI database.

About the accuracy of the method, the presence of ACTB was found in all the positive samples (*n* = 1002). Therefore, the diagnostic sensitivity was 100% (95% CI: 99.6–100%).

Diagnostic specificity was evaluated as the ratio between the number of samples, in which the presence of ACTB was not expected (here, all the serum samples tested, *n* = 40), and the number of all samples tested resulted negatives (here, the sum of all serum samples tested as negative and serum samples eventually resulted as positive, *n* = 40 + 0). Therefore, the diagnostic specificity was 100% (95% CI: 91.2–100%).

Regarding the reproducibility, the two operators obtained the same results analyzing the same 48 samples. Furthermore, 48 samples were also amplificated using two different thermocyclers, and the results were the same for both instruments.

Then the Cohen’s kappa for the three categorical results (WNS/USUV negative and ACTB positive; two WNS/USUV positive and ACTB positive; and one WNS/USUV negative and ACTB negative) was equal to 1 (95% CI: 0.77–1.23).

The limit of detection (LOD) of the method was assessed as follows: for West Nile virus Lineage 1, the LOD was 10^1.8^ TCID_50_/mL; for West Nile virus Lineage 2, the LOD was 10^0.5^ TCID_50_/mL; and for Usutu virus, the LOD was 10^1.72^ TCID_50_/mL.

## 4. Discussion and Conclusions

The development of biomolecular methods for the detection of mosquito-borne viruses, such as West Nile virus and Usutu virus, is critical for public health from a One Health perspective. These viruses, which can cause severe diseases in humans and animals, are increasingly recognized as significant threats in the context of climate change, global travel, and the expansion of mosquito habitats.

Following the key point reported in the PNA, robust and accurate detection methods are essential to enable timely surveillance, outbreak prediction, and rapid response. Early identification of these viruses in mosquito populations, wildlife, or human beings helps to mitigate the risk of widespread transmission and facilitates targeted vector control measures.

Among all the zoonotic diseases of public health importance, the emergence and spread of these arboviruses highlights the intricate connections between human beings, animals and environmental health. A One Health approach emphasizes the need for integrated biomolecular diagnostics that can monitor viral circulation across different species and ecological settings. Such methods could enhance our ability to detect spillover events and manage the risk of zoonoses, safeguarding both human and animal populations. Despite the advantages of endogenous controls, there is a notable lack of the literature on their use as quality controls in molecular analyses of vector arthropods, especially in arboviruses detection such as WNV and USUV. Instead, commercially produced exogenous genes are commonly used. In this study, we reported the use of the endogenous ACTB gene as a robust quality control in co-amplification reactions with these viruses. As reported in the literature, housekeeping genes, which are naturally present within a sample, offer a more reliable internal control compared to exogenous controls: they verify extraction efficiency, accounting for variations in sample processing and potential inhibitors.

However, co-amplification with the target gene can be problematic, potentially leading to competition for resources like dNTPs and polymerase: a proper balance between the reagents is needed to permit amplification of the endogenous control without negatively impacting the amplification of the target gene. An additional innovative aspect of our study was the use, in field-collected mosquito pools, of degenerate primers along with a single probe, which had already been described in the literature for the detection of ACTB mRNA in various vertebrate and invertebrate cell lines, including mosquito-derived cell lines [18]. No data have been reported regarding the use of these primers in studies on field samples before the present work.

In our research, we implemented a duplex RT-PCR approach, combining the use of degenerate primers for detecting ACTB mRNA—which served as a housekeeping gene in mosquitoes—with an established molecular assay for the identification of WNV and USUV [19], already in use in our laboratory for national mosquito-borne pathogen surveillance. This strategy allowed us to integrate the detection of mosquito RNA integrity with the simultaneous identification of both viruses in a single reaction and demonstrated excellent accuracy and reproducibility. This approach also provided a robust mechanism to verify RNA extraction quality and the absence of PCR inhibitors.

The results demonstrated 100% diagnostic sensitivity and specificity in distinguishing positive and negative samples, underscoring the assay’s reliability for large-scale arbovirus surveillance. Importantly, the amplification of the ACTB mRNA was exclusive for mosquito pools, with no significant cross-reactivity observed in horse or bovine serum samples, validating the primers’ specificity for the reaction. Sanger sequencing further confirmed the identity of the amplified ACTB region, corroborating the method’s precision across various mosquito species.

The duplex Real-Time RT-PCR approach effectively identified the target viruses and the endogenous mRNA in all six pools positive for WNV or USUV. Moreover, the statistical approach applied in sample size selection ensured that the results are consistent not only under controlled conditions but also when minor variations in operation and equipment occur (reproducibility). This approach provides confidence in the method’s reliability for broader applications.

In future applications, we aim to extend this protocol to vector arthropod fragments. The LOD of this method in fact allows the detection of only a few gene copies, demonstrating its high analytical sensitivity. These findings confirm that the developed duplex RT-PCR assay can detect viral RNA even at concentrations lower than those typically required by the National Reference Centre during interlaboratory proficiency tests, further supporting its robustness and reliability.

In conclusion, the integration of degenerate primers for the ACTB mRNA into a duplex Real-Time RT-PCR assay represents a significant advancement in arbovirus surveillance methodologies. This approach establishes a foundation for further optimization and application of biomolecular assays in public health surveillance, ensuring timely detection and response to emerging arboviral threats.

## Figures and Tables

**Table 1:** Mosquito sampling by species and provinces of Piedmont region.

Species	Alessandria		Asti		Biella		Cuneo		Novara		Turin		Verbania		Vercelli		Total	
	*n*	Pool	*n*	Pool	*n*	Pool	*n*	Pool	*n*	Pool	*n*	Pool	*n*	Pool	*n*	Pool	*n*	Pool
*Aedes albopictus*	491	24	9	4	26	11	67	14	79	8	761	50	55	10	51	9	1539	130
*Aedes cinereus*	0	0	0	0	0	0	0	0	0	0	0	0	4	1	0	0	4	1
*Aedes japonicus*	0	0	0	0	1	1	0	0	0	0	0	0	0	0	0	0	1	1
*Aedes vexans*	9	3	0	0	0	0	19	3	13	4	101	14	0	0	3	1	145	25
*Anopheles maculipennis*	168	15	0	0	0	0	0	0	905	16	18	3	0	0	1116	35	2207	69
*Anopheles plumbeus*	4	1	2	2	3	2	0	0	0	0	3	3	0	0	1	1	13	9
*Culex modestus*	180	9	61	3	0	0	0	0	186	7	8	1	0	0	31	6	466	26
*Culex pipiens*	3825	88	1377	50	112	22	2145	75	1623	37	2285	89	81	15	1789	58	13,237	434
*Culiseta annulata*	0	0	0	0	0	0	0	0	0	0	0	0	0	0	0	0	0	0
*Culiseta longiareolata*	3	2	1	1	2	2	0	0	0	0	0	0	0	0	0	0	6	5
*Culiseta subochrea*	0	0	0	0	0	0	0	0	0	0	0	0	0	0	0	0	0	0
*Ochlerotatus cantans*	0	0	0	0	0	0	0	0	0	0	1	1	32	5	0	0	33	6
*Ochlerotatus caspius*	4265	84	1410	39	834	17	48	13	2853	42	788	37	98	3	2212	56	12,508	291
*Ochlerotatus geniculatus*	1	1	0	0	0	0	1	1	0	0	2	2	0	0	1	1	5	5
**Total**	8946	227	2860	99	978	55	2280	106	5659	114	3967	200	270	270	5204	167	30,164	1002

**Table 2:** Primers and probes for the duplex Real-Time RT-PCR for ACTB mRNA and a common region of 92 bp for WNV and USUV.

Primer and Probes		Sequences
Forward primer	WNTang Fw	AAGTTGAGTAGACGGTGCTG
Reverse primer	WNTang Rw	AGACGGTTCTGAGGGCTTAC
Probe (FAM-BHQ1)	WNTang Probe	CTCAACCCCAGGAGGACTGG
Forward primer	ACTB Fw	GTSTGGATYGGHGGHTCBATC
Reverse primer	ACTB Rw	GAYTCRTCRTAYTCCTSCTTG
Probe (VIC-TAMRA)	ACTB Probe	ACCTTCCAGCAGATGTGGATC

**Table 3:** Thermocycling Profile for the duplex Real-Time RT-PCR for ACTB mRNA and a common region of 92 bp for WNV and USUV.

Phase	Temperature (°C)	Duration	Number of Cycles
Reverse transcription	50	30 min	1
Initial denaturation	95	15 min	1
Denaturation	95	30 s	45
Annealing	60	60 s	

**Table 4:** Primers and probes for the duplex Real-Time RT-PCR able to detect WNV Lineage 1 and Lineage 2 and for the simplex Real-Time RT-PCR able to detect USUV.

Primers and Probes	Sequences
WNV F primer	GTGATCCATGTAAGCCCTCAGAA
WNV R primer	GTCTGACATTGGGCTTTGAAGTTA
WNV Lineage 1 probe	AGGACCCCACATGTT
WNV Lineage 2 probe	AGGACCCCACGTGCT
USUTU F primer	AAAAATGTACGCGGATGACACA
USUTU R primer	TTTGGCCTCGTTGTCAAATC
USUTU probe	CGGCTGGGACACCCGGATAACC

## Data Availability

The data presented in this study are available on request from the corresponding author. The data are not publicly available due to privacy.

## Abbreviations

The following abbreviations are used in this manuscript:

WNV	West Nile Virus
USUV	Usutu Virus
ECDC	European Centre for Disease Prevention and Control
PNA	Piano Nazionale Arbovirosi–Arboviroses National Plan
TBE	Tick-Borne Encephalitis
TOSV	Toscana Virus
(m)RNA	(messenger)Ribonucleic Acid
(RT)PCR	(Real-Time) Polymerase Chain Reaction
ACTB	β-actin
CESME	National Reference Centre for Arboviroses
RLT	RNA Lysis Buffer
dNTPs	Deoxynucleotides
